# A Systematic Review to Assess Gender Diversity in Authorship Within the Orthopaedic Surgery Literature

**DOI:** 10.1007/s43465-025-01462-x

**Published:** 2025-07-02

**Authors:** Bryn O. Zomar, Tarini Boparai, Erin You, Kendra Jackson, Natalie South, Emily K. Schaeffer

**Affiliations:** 1 Department of Orthopaedics, University of British Columbia, Vancouver, BC Canada; 2 Department of Orthopaedic Surgery, BC Children’s Hospital, 1D.18-4480 Oak Street, Vancouver, BC V6H 3V4 Canada

**Keywords:** Gender diversity, Systematic review, Orthopaedic surgery, Authorship

## Abstract

**Background:**

Gender diversity trends in orthopaedic research are dynamic. While an increase of women in orthopaedics has been observed, gender imbalances continue to exist, especially in academic leadership and research roles. The purpose of our study was to assess the representation of women in authorship roles over a 20-year period.

**Methods:**

We conducted a systematic review of clinical research studies published in The Journal of Bone and Joint Surgery and The Bone and Joint Journal between 1996–2000 and 2016–2020. First, corresponding and last author gender was determined using a combination of automated name analysis and manual searches. We performed chi-squared tests to assess differences in the proportion of women in each authorship position across time periods, journals, and orthopaedic subspecialties.

**Results:**

Women represented 12.4% of first authors, 8.1% of last authors and 10.5% of corresponding authors. The proportion of women in first and corresponding author positions increased over time (*p* < 0.001 and *p* < 0.001 respectively) while there was no difference for last author position (*p* = 0.572). No differences were found when comparing last authors across the subspecialties (*p* = 0.149 respectively); however, there was a difference for first and corresponding authors (*p* = 0.019 and *p* = 0.024 respectively), with the highest proportion of women reported in general orthopaedics (19.0% and 17.7% respectively) and lowest in sports medicine (8.1%) and lower extremity (6.6%).

**Conclusion:**

This study found improvements in the representation of women in first and corresponding author roles, however significant gaps remain, particularly in leadership positions represented by last author position. Continued monitoring and intervention are essential to promote long-term, meaningful change in the field.

## Introduction

Equity, diversity, and inclusion (EDI) are increasingly recognized as essential principles in medical research, yet disparities in authorship representation persist. Orthopaedics has been identified as one of the least diverse medical specialties, with a particularly low representation of women and other minority groups in both clinical and academic settings [1]. Despite the growing presence of women in orthopaedics, it remains a primarily male-dominated field, with a continued pattern of gender imbalances in academic leadership positions [2]. Previous studies have highlighted persistent disparities in authorship within orthopaedic literature, consistently showing that women remain underrepresented as first and senior authors [3]. This underrepresentation carries broader implications for orthopaedic research and limits the diversity of perspectives that drive innovation and clinical advancements.

Authorship trends and research collaboration patterns can reflect systemic inequities within the field. Although recent studies have documented the persistent gender gap in medical research, there remains a lack of comprehensive analyses that track these trends over time, specifically in orthopaedic surgery. While some studies have examined gender disparities in general medicine, research on authorship trends in orthopaedic journals remains limited, making it difficult to assess the extent of the gender gap in this field [4].

The primary aim of this bibliometric analysis study was to assess gender diversity in orthopaedic authorship in two prominent orthopaedic journals over a 20-year time span.

## Methods

To assess the involvement of women in authorship positions in the orthopaedic literature, we conducted a systematic review of studies published in two leading orthopaedic journals: the *Bone and Joint Journal* (BJJ) (formerly known as the *Journal of Bone and Joint Surgery, British volume*), and the *Journal of Bone and Joint Surgery* (JBJS) (formerly known as the *Journal of Bone and Joint Surgery, American volume*).

Studies eligible for inclusion were those published in either journal between 1996–2000 and 2016–2020. We included clinical research studies, such as randomized controlled trials, cohort studies, case–control studies, and chart reviews. Studies published outside the designated time frames were excluded, as were non-clinical publications such as editorials, memorandums, letters to the editor, commentaries, reviews, etc. We also excluded case reports. Further, any study published online but not yet printed in the final volume of the journal within the specified date range was also excluded. Finally, studies involving animals, cadavers, or simulation studies were also excluded.

Eligible publications were reviewed, and relevant bibliographic data was extracted using REDCap [5, 6]. We collected the year of publication, journal name, study title, first and last name for first, last and corresponding authors. The position of the corresponding author on the byline was also noted. If a study had a single listed author, they were designated as the first author and no last author was noted. Each study was also classified by subspecialty: Arthroscopy, Arthroplasty, Trauma, Oncology, Sports, Pediatrics, Spine, Lower Extremity, Upper Extremity or General Orthopaedics. Lower extremity included all studies related to the hip, knee, foot and ankle that did not fall under any of the other subspecialty categories, similar to upper extremity which included all studies related to shoulder, elbow, hand and wrist. We categorized studies under general orthopaedics if they did not fall under any of the other specified subspecialty categories.

We also collected the gender of the first, last and corresponding authors. Gender was determined using the protocol described in Squire et al. and Fisher et al., adapted from the methodology in Feramisco et al. [7–9]. To assess authors’ genders, we used Geoff Peters’ Baby Name Guesser (https://www.gpeters.com/names/baby-names.php), a website that assigns a gender and gender ratio to first names. If the gender ratio was 3.0 or higher, the assigned gender was presumed to be correct. For names with a ratio below 3.0, a manual search was conducted to identify gender-specific information about the author. If the search was inconclusive or if a first name could not be identified, the gender was recorded as unknown.

We performed a series of chi-squared tests to assess whether the proportion of women in each authorship position, first, last or corresponding, differed significantly across several factors. We compared between the time periods, between the journals, and across the orthopaedic subspecialties. Within each journal and each subspecialty, we also assessed whether the proportion of women in each authorship position differed over time. All analyses excluded when gender was unknown. Comparisons were deemed to be significant at a level of *p* = 0.05.

## Results

Our search returned a total of 6011 papers, of which 3137 met our eligibility criteria. A total of 1720 (54.8%) papers were published in BJJ and 1417 (45.2%) were published in JBJS (Table 1). The total number of papers published increased by 62.7% between the time periods with 1209 (38.5%) published between 1996–2000 and 1928 (61.5%) between 2016 and 2020. We were unable to determine the gender for a combined 4.1% of reported corresponding, first and last authors.

**Table 1:** Article distribution by journal and publication year

Year published	Journal	
	BJJ	JBJS
1996–2000	658	551
2016–2020	1062	866

**Fig. 1:**
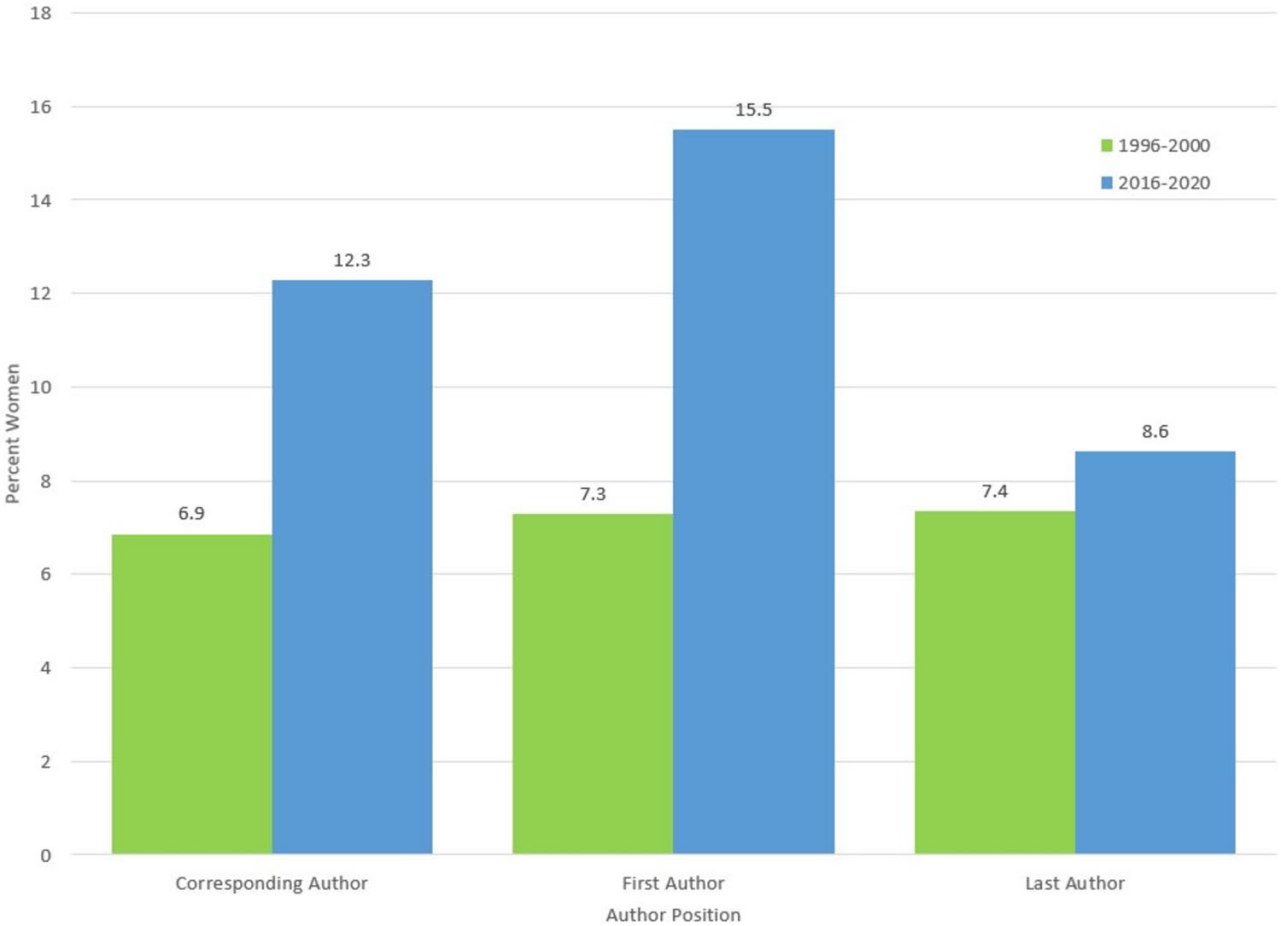
Percentage of women as corresponding, first and last authors over time

**Fig. 2:**
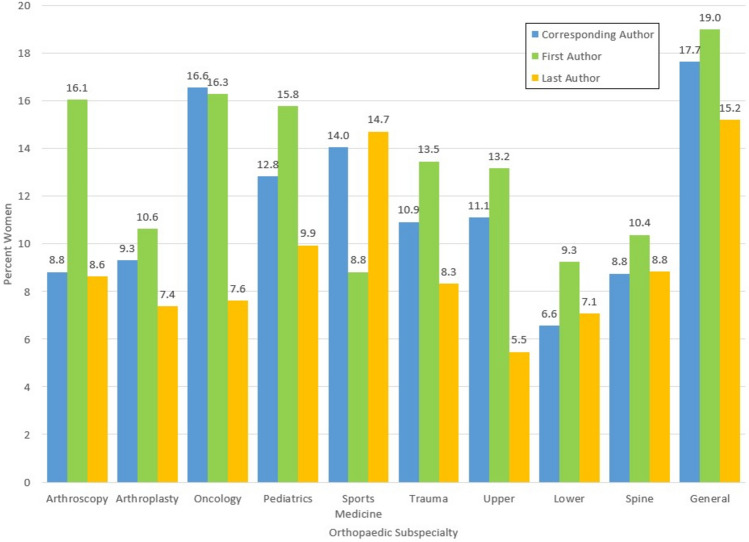
Percentage of women as corresponding, first and last authors in each of the orthopaedic subspecialties

**Fig. 3:**
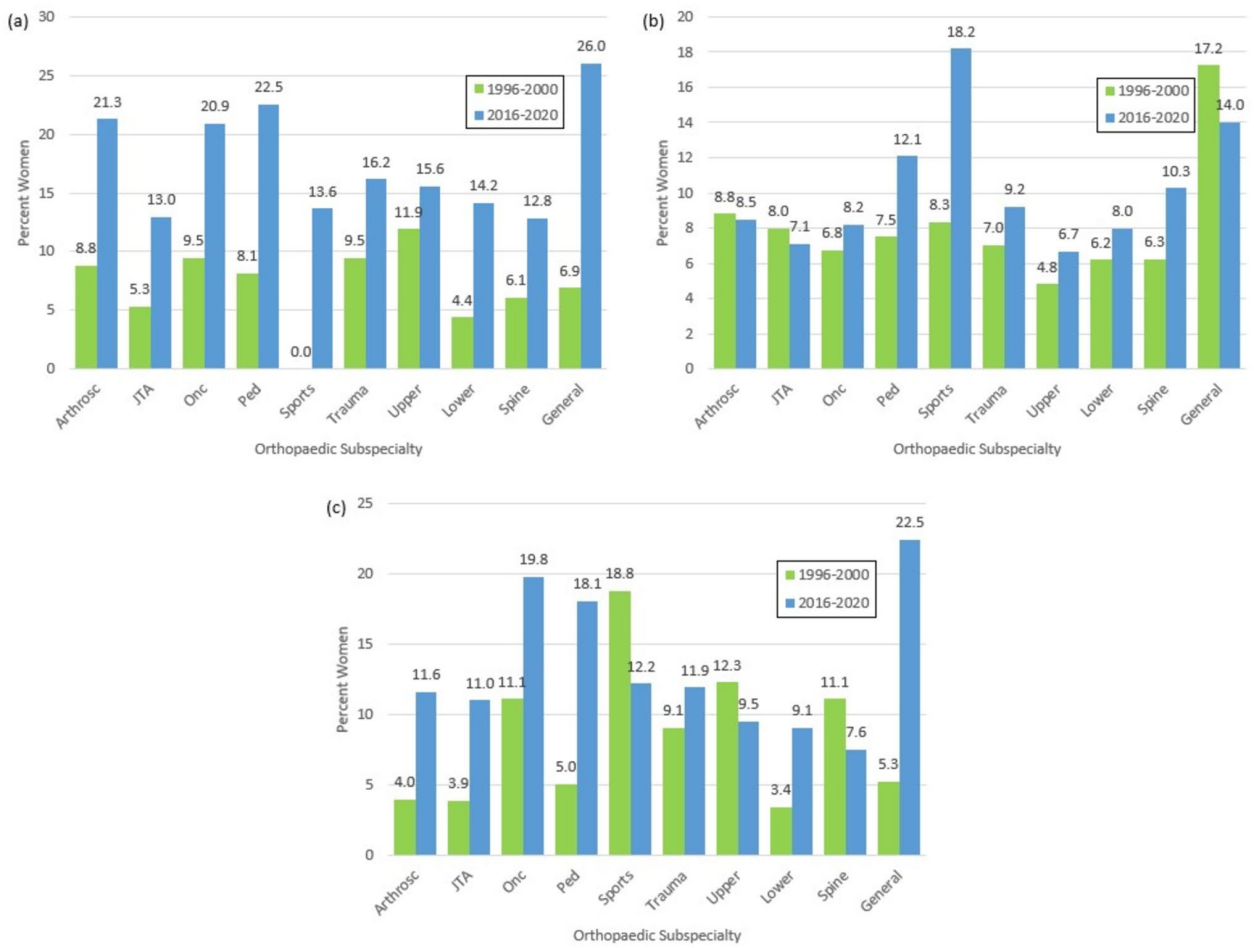
Percentage of women as **a** first, **b** last, and **c** corresponding authors over time in each of the orthopaedic subspecialties. Arthrosc = Arthroscopy; JTA = Arthroplasty; Onc = Oncology; Ped = Pediatrics; Sports = Sports Medicine

Overall, 12.4% of first authors were women and the proportion significantly differed between 1996–2000 and 2016–2020 (7.3% vs. 15.6%, *p* < 0.001) (Table 2; Fig. 1). The same trend was also significant when comparing the time periods separately for each journal (*p* < 0.001 and *p* < 0.001 respectively). The proportion of women reported as first authors also differed significantly when comparing across the orthopaedic subspecialties (*p* = 0.019) with general orthopaedics reporting the highest proportion of women as first authors (19.0%) and sports medicine the lowest (8.1%) (Fig. 2). When comparing the time periods for each subspecialty individually, the proportion of women reported as first authors was significantly different for arthroplasty (*p* < 0.001), oncology (*p* = 0.029), pediatrics (*p* = 0.001), trauma (*p* = 0.033), lower extremity (*p* = 0.013) and general orthopaedics (*p* = 0.033) (Fig. 3a).

**Table 2:** Distribution of the gender of authors in the first author position

	First author gender							*p* value
	Female	%	Male	%	Unknown	%	Total	
Overall	388	12.37	2623	83.61	126	4.02	3137	
Years								
1996–2000	88	7.33	1046	86.52	75	6.20	1209	**<0.001**
2016–2020	300	15.56	1577	81.79	51	2.65	1928	
Journal								
BJJ	202	11.74	1429	83.08	89	5.17	1720	0.372
JBJS	186	13.13	1194	84.26	37	2.61	1417	
BJJ								
1996–2000	48	7.29	547	83.13	63	9.57	658	**<0.001**
2016–2020	154	14.50	882	83.05	26	2.45	1062	
JBJS								
1996–2000	40	7.26	499	90.56	12	2.18	551	**<0.001**
2016–2020	146	16.86	695	80.25	25	2.89	866	
Subspecialties								
Arthroscopy	13	16.05	65	80.25	3	3.70	81	**0.019**
Arthroplasty	133	10.63	1081	86.41	37	2.96	1251	
Oncology	30	16.30	145	78.80	9	4.89	184	
Pediatrics	54	15.79	266	77.78	22	6.42	342	
Sports Medicine	6	8.13	60	88.24	2	2.94	68	
Trauma	91	13.46	553	81.80	32	4.73	676	
Upper Extremity	17	13.18	106	82.17	6	4.65	129	
Lower Extremity	21	9.25	199	87.67	7	3.08	227	
Spine	19	15.63	155	78.13	9	6.25	183	
General	15	18.99	63	79.75	1	1.27	79	
Arthroscopy								
1996–2000	3	8.82	29	85.29	2	5.88	34	0.150
2016–2020	10	21.28	36	76.60	1	2.13	47	
Arthroplasty								
1996–2000	20	5.28	336	88.65	23	6.07	379	**<0.001**
2016–2020	113	12.96	745	85.44	14	1.61	872	
Oncology								
1996–2000	7	9.46	65	87.84	2	2.70	74	**0.029**
2016–2020	23	20.91	80	72.73	7	6.26	110	
Pediatrics								
1996–2000	13	8.13	131	81.88	16	10.00	160	**0.001**
2016–2020	41	22.53	135	74.18	6	3.30	182	
Sports Medicine								
1996–2000	0	0	23	95.83	1	4.17	24	0.060
2016–2020	6	13.64	37	84.09	1	2.27	44	
Trauma								
1996–2000	26	9.49	228	83.21	20	7.30	274	**0.033**
2016–2020	65	16.17	325	80.85	12	2.99	402	
Upper Extremity								
1996–2000	10	11.90	70	83.33	4	4.76	84	0.563
2016–2020	7	15.56	36	80.00	2	4.44	45	
Lower Extremity								
1996–2000	5	4.39	104	91.23	5	4.39	114	**0.013**
2016–2020	16	14.16	95	84.07	2	1.77	113	
Spine								
1996–2000	4	6.06	59	89.39	3	4.55	66	0.145
2016–2020	15	12.82	96	82.05	6	5.13	117	
General								
1996–2000	2	6.90	27	93.10	0	0	29	**0.033**
2016–2020	13	26.00	36	72.00	1	2.00	50	

Bold values indicate statistically significant *p*-values (*p* < 0.05)

In total, 8.1% of last authors were women (Table 3). There was no difference in the proportion of women compared between the time periods (*p* = 0.572) (Fig. 1), however there was a difference when comparing between the journals (*p* = 0.027). When looking at each journal independently and comparing across the time periods, there was a significant difference for JBJS (*p* = 0.035) but not for BJJ (*p* = 0.196). There was no difference in the proportion of women reported as last author when comparing across the orthopaedic subspecialties (*p* = 0.149) (Fig. 2) or when comparing between the time periods within each subspecialty (*p* > 0.05) (Figs. 3b).

**Table 3:** Distribution of the gender of authors in the last author position

	Last author gender							*p* value
	Woman	%	Man	%	Unknown	%	Total	
Overall	254	8.13	2715	86.91	155	4.96	3124	
Years								
1996–2000	88	7.35	989	82.55	120	10.10	1198	0.572
2016–2020	166	8.62	1726	89.62	34	1.77	1926	
Journal								
BJJ	119	6.97	1468	86.00	120	7.03	1707	**0.027**
JBJS	135	9.53	1247	88.00	35	2.47	1417	
BJJ								
1996–2000	47	7.26	494	76.35	106	16.38	647	0.196
2016–2020	72	6.79	974	91.89	14	1.32	1060	
JBJS								
1996–2000	41	7.44	495	89.84	15	2.72	551	**0.035**
2016–2020	94	10.85	752	86.84	20	2.31	866	
Subspecialties								
Arthroscopy	7	8.64	71	87.65	3	3.70	81	0.149
Arthroplasty	92	7.39	1107	88.92	46	3.69	1245	
Oncology	14	7.61	162	88.04	8	4.35	184	
Pediatrics	34	9.94	285	83.33	23	6.73	342	
Sports Medicine	10	14.71	54	79.41	4	5.88	68	
Trauma	56	8.32	575	85.44	42	6.24	673	
Upper Extremity	7	5.47	113	88.28	8	6.25	128	
Lower Extremity	16	7.08	197	87.17	13	5.75	226	
Spine	16	8.84	155	85.64	10	5.52	181	
General	12	15.19	66	83.64	1	1.27	79	
Arthroscopy								
1996–2000	3	8.82	28	82.35	3	8.82	34	0.860
2016–2020	4	8.51	43	91.49	0	0.00	47	
Arthroplasty								
1996–2000	30	8.00	309	82.40	36	9.60	375	0.337
2016–2020	62	7.13	798	91.49	10	1.15	870	
Oncology								
1996–2000	5	6.76	64	86.49	5	6.76	74	0.780
2016–2020	9	8.18	98	89.09	3	2.73	110	
Pediatrics								
1996–2000	12	7.50	126	78.75	22	13.75	160	0.321
2016–2020	22	12.09	159	87.36	1	0.55	182	
Sports Medicine								
1996–2000	2	8.33	19	79.17	3	12.50	24	0.348
2016–2020	8	18.18	35	79.55	1	2.27	44	
Trauma								
1996–2000	19	7.01	219	80.81	33	12.18	271	0.540
2016–2020	37	9.20	356	88.56	9	2.24	402	
Upper Extremity								
1996–2000	4	4.82	75	90.36	4	4.82	83	0.617
2016–2020	3	6.67	38	84.44	4	8.89	45	
Lower Extremity								
1996–2000	7	6.19	93	82.30	13	11.50	113	0.790
2016–2020	9	8.96	104	92.04	0	0	113	
Spine								
1996–2000	4	6.25	56	87.50	4	6.25	64	0.375
2016–2020	12	10.26	99	84.62	6	5.13	117	
General								
1996–2000	5	17.24	23	79.31	1	3.45	29	0.651
2016–2020	7	14.00	43	86.00	0	0	50	

Bold values indicate statistically significant *p*-values (*p* < 0.05)

At least one corresponding author was indicated in 86.5% of papers and more often in those published between 2016–2020 (94.7%) than 1996–2000 (73.5%) (Table 4). Overall, 10.5% of corresponding authors were women and the proportion differed significantly between the time periods (*p* < 0.001) (Fig. 1). The difference was also significant when looking at each journal individually (*p* = 0.005 and *p* = 0.006 respectively). There was also a difference in the proportion of women corresponding authors across the subspecialties (*p* = 0.024) with general orthopaedics reporting the highest proportion of women as corresponding authors (17.7%) and lower extremity the lowest (6.6%) (Fig. 2). When comparing between the time periods, there was a significant difference when looking at specific orthopaedic subspecialties including arthroplasty (*p* = 0.002) and pediatrics (*p* = 0.006) (Fig. 3c).

**Table 4:** Distribution of the gender of authors indicated as the corresponding author

	Corresponding author gender							*p* value
	Woman	%	Man	%	Unknown	%	Total	
Overall	285	10.50	2343	86.33	86	3.17	2714	
Years								
1996–2000	61	6.86	777	87.40	51	5.74	889	**<0.001**
2016–2020	224	12.27	1566	85.81	35	1.92	1825	
Journal								
BJJ	156	9.83	1372	86.45	59	3.72	1587	0.217
JBJS	129	11.45	971	86.16	27	2.40	1127	
BJJ								
1996–2000	46	6.99	563	85.56	49	7.45	658	**0.005**
2016–2020	110	11.84	809	87.08	10	1.08	929	
JBJS								
1996–2000	15	6.49	214	92.64	2	0.87	231	**0.006**
2016–2020	114	12.72	757	84.49	25	2.79	896	
Subspecialties								
Arthroscopy	6	8.82	61	89.71	1	1.47	68	**0.024**
Arthroplasty	100	9.30	949	88.28	26	2.42	1075	
Oncology	28	16.57	135	79.88	6	3.55	169	
Pediatrics	38	12.84	247	83.45	11	3.72	296	
Sports Medicine	8	14.04	46	80.70	3	5.26	57	
Trauma	65	10.92	501	84.20	29	4.87	595	
Upper Extremity	11	11.11	88	88.89	0	0	99	
Lower Extremity	13	6.57	182	91.92	3	1.52	198	
Spine	14	8.75	139	86.88	7	4.38	160	
General	12	17.65	55	80.88	1	1.47	68	
Arthroscopy								
1996–2000	1	4.00	23	92.00	1	4.00	25	0.305
2016–2020	5	11.63	38	88.37	0	0	43	
Arthroplasty								
1996–2000	10	3.88	234	90.70	14	5.43	258	**0.001**
2016–2020	90	11.02	715	87.52	12	1.47	817	
Oncology								
1996–2000	7	11.11	55	87.30	1	1.59	63	0.118
2016–2020	21	19.81	80	75.47	5	4.72	106	
Pediatrics								
1996–2000	6	5.04	104	87.39	9	7.56	119	**0.002**
2016–2020	32	18.08	143	80.79	2	1.13	177	
Sports Medicine								
1996–2000	3	18.75	11	68.75	2	12.50	16	0.418
2016–2020	5	12.20	35	85.75	1	2.44	41	
Trauma								
1996–2000	19	9.09	170	81.34	20	9.57	209	0.450
2016–2020	46	11.92	331	85.75	9	2.33	386	
Upper Extremity								
1996–2000	7	12.28	50	87.72	0	0	57	0.666
2016–2020	4	9.52	38	90.48	0	0	42	
Lower Extremity								
1996–2000	3	3.41	82	93.18	3	3.41	88	0.123
2016–2020	10	9.09	100	90.91	0	0	110	
Spine								
1996–2000	6	11.11	46	85.19	2	3.70	54	0.462
2016–2020	8	7.55	93	87.74	5	4.72	106	
General								
1996–2000	1	5.26	18	94.74	0	0	19	0.089
2016–2020	11	22.45	37	75.51	1	2.04	49	

Bold values indicate statistically significant *p*-values (*p* < 0.05)

**Fig. 4:**
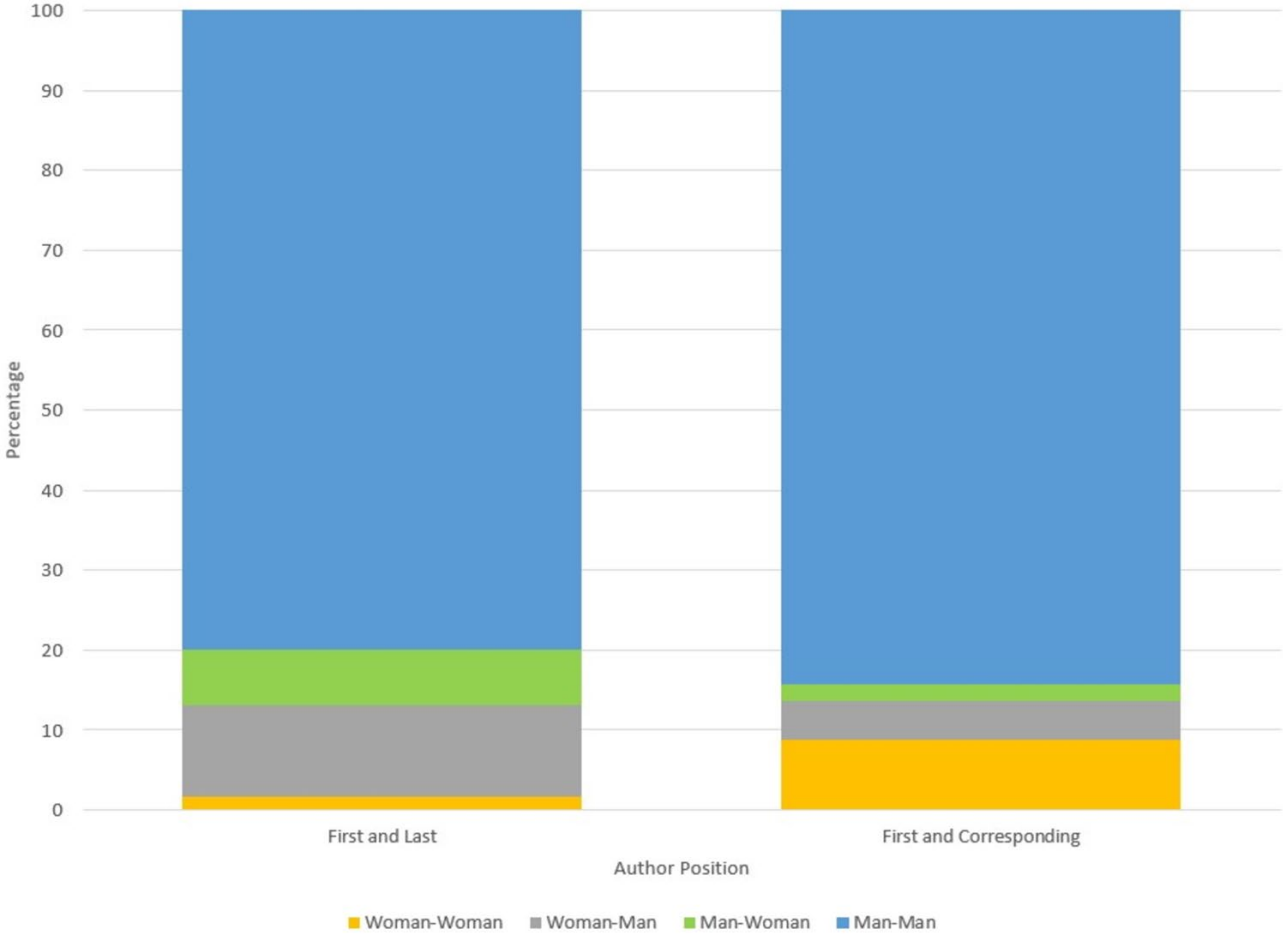
Combinations of gender of first and last or first and corresponding authors

Of the 2894 papers which had more than one author listed, and gender was able to be determined, 46 (1.6%) had a woman as both the first and last author (Fig. 4). And for papers which had a corresponding author listed, 227 (8.8%) had a woman as both the first and corresponding author.

## Discussion

Our study demonstrates that women’s representation in the authorship of orthopaedic publications has improved over time, however, a substantial gender gap remains. Across the analyzed time periods and journals, men continue to dominate authorship, with women accounting for only an average of 10% of authors. This unequal gender distribution highlights the persistent underrepresentation of women in orthopaedic research authorship.

Our analyses found women comprise only 10.5% of corresponding authors, 12.4% of first authors and 8.2% of last authors, compared to 86.3%, 83.7% and 86.9% of men, respectively. A study by Brown et al. that examined women’s authorship in six major orthopaedic journals from 1987 to 2017, found that while women’s authorship has risen over time, this increase has been notably slower than the parallel rise in the number of women entering the profession [3]. Interestingly, when comparing our findings with workforce demographics, where women represent roughly 4.6% of orthopaedic surgeons globally [10], representation of women in all authorship positions was actually higher than might be expected. Although women remain underrepresented in academic orthopaedics, the authorship rates we found may suggest that women are more prolific in publishing research than their counterparts. While this could explain the higher-than-expected authorship proportions, it was not an area directly examined in our analysis.

When comparing between the time periods, 1996–2000 and 2016–2020, we observed a significant increase in women’s representation in authorship, particularly in the corresponding and first author positions. In contrast, the last author position, often indicative of seniority and leadership within research teams [11–13], did not see a significant improvement in women’s representation. This trend aligns with findings from other studies [14, 15]. The rise in women’s involvement in authorship is encouraging and may be attributed to the growing number of women entering the orthopaedic surgery profession. However, the slower growth in last author positions likely reflects barriers to academic progression, as achieving senior roles typically requires additional years of experience, mentorship, and institutional support [16–18]. This disparity is further corroborated by data showing that women in surgical faculty positions remain disproportionately concentrated in lower academic ranks [19, 20]. Although women constitute 17.8% of the academic faculty in orthopaedic surgery in the US, only 8.7% hold professorships, with the majority occupying the lower rank of instructor [20]. Furthermore, as of the 2015–2016 academic year, only one woman had attained the position of orthopaedic surgery department chair [20]. In the UK, while representation of women in orthopaedic surgery positions has continued to increase, in 2020, only 25% of trainees and 7% of consultants were women [21].

Our analysis revealed that the proportion of women in the first author position differed significantly across the orthopaedic subspecialties. Notably, pediatric orthopaedics had one of the highest proportion of female first authors, while sports medicine had the lowest. This pattern aligns with previous research indicating that pediatric orthopaedics is a relatively more gender diverse subspecialty [22, 23], whereas sports medicine continues to have lower female representation [24–26]. In contrast to the first author position, we did not find a difference across the subspecialties for senior author position (last). This is rather surprising given that previous research has demonstrated that female leadership within pediatric orthopaedics is also comparatively higher than other subspecialties, with women leading 13.3% of pediatric orthopaedic fellowship programs in 2021 [27]. In contrast, sports medicine has the lowest female representation in leadership, with women heading just 2.2% of fellowship programs in the same year [27].

This study has several limitations. Although only 4% of authors’ gender was ultimately unknown, a lack of available information to determine the gender of authors presented a significant challenge during data collection. This issue was especially prominent in papers published during the 1996–2000 time period. During searches to identify these authors, details such as first name, position or affiliation were often publicly unavailable or difficult to find. This is not surprising as there was limited digitization and thus, less linkage of author information during this time. Additionally, while the JBJS typically includes authors’ first names in the headings of their publications, the BJJ continues to publish only authors’ first initials, further complicating the ability to accurately assess gender. While we followed established guidelines from previous research to determine author gender [7–9], there may have been misclassifications in gender, particularly for authors with non-traditional first names. Gender determination by name alone can be inaccurate, as it ultimately depends on the researcher’s or author’s self-identification. Nevertheless, this approach remains the best available option until journals begin mandating the inclusion of authors’ demographic information and making it publicly accessible. Moreover, our analysis focused only on those indicated as the corresponding author and those in the first and last author positions. We did not include the remaining middle authors in our analyses, many of whom are likely women, however the author positions we did include generally represent those who contribute the majority of the work for each publication. Another limitation was the inclusion of only two journals in this analysis. It’s possible that more sub-specialty focused journals may show different trends in authorship and therefore more research that delves specifically into each subspecialty is warranted.

Our study highlights the importance of increasing mentorship and leadership opportunities for women in orthopaedics to facilitate their advancement into leadership roles and ultimately senior authorship position. Institutions and funding bodies should prioritize initiatives that support women at early and mid-career stages, to help overcome the barriers that currently hinder their progression to senior positions. Notably, research within the field of orthopaedics has shown that women benefit significantly from same-gender mentorship [28], underscoring the need to expand and foster mentorship programs led by senior women in the field to increase women’s representation. By creating a more inclusive and supportive environment, we can ensure that women’s contributions to orthopaedic research continues to grow, ultimately leading to a more diverse and equitable field. Over time, we hope to see a continued increase in female author contribution to the orthopaedic literature in proportion to the rise in participation of women in the orthopedic surgery specialty.

## Data Availability

The datasets generated and/or analyzed during the current study are available from the corresponding author on reasonable request.
